# Social-Emotional Development in the Summer Art Camp Setting

**DOI:** 10.7759/cureus.65448

**Published:** 2024-07-26

**Authors:** Kathryn C Lotharius, Clara A Finley, Peter Averkiou

**Affiliations:** 1 Integrated Medical Science, Florida Atlantic University Charles E. Schmidt College of Medicine, Boca Raton, USA; 2 Education, Lake Forest College, Lake Forest, USA

**Keywords:** school-aged children, casel framework, art camp, summer camp, social-emotional learning, social-emotional development

## Abstract

Introduction

Social-emotional development refers to the development of one's abilities to understand, regulate, and express emotions and to establish and maintain successful relationships with peers and adults. Education in the arts has been shown to promote learning these skills, but the relationship between social-emotional development and summer art camp has not been explored.

Methods

The objective of this study is to determine the potential for social-emotional development in a community-based art day camp. A qualitative thematic analysis of the art camp's curriculum was conducted and compared with current literature regarding opportunities for social-emotional development in arts education and summer camp settings.

Results

The summer art camp curriculum included practices known to facilitate social-emotional learning in school-aged children. The curriculum data themes identified were performance, art projects, and outdoor activities. All of these themes have been shown to facilitate social-emotional skill building and can be connected to the components of the Collaborative for Academic, Social, and Emotional Learning (CASEL) framework.

Conclusions

Through the shown benefits of summer camp in combination with the benefits of in-school arts education, art camp provides the unique opportunity to practice self-expression, friend-making, and self-esteem building, all of which can contribute to mental well-being and academic success long-term.

## Introduction

Social-emotional development (SED) refers to the development of one's abilities to understand, regulate, and express emotions and to establish and maintain successful relationships with peers and adults [1]. In recent years, there has been an increased focus on social-emotional learning (SEL) in K-12 schools in the United States, as demonstrated by the growing number of state standards aimed at cultivating these skills [2]. SEL refers to the skill-building practices that contribute to SED. The skills associated with SEL, as outlined by the Collaborative for Academic, Social, and Emotional Learning (CASEL) framework commonly used in schools, are self-management, self-awareness, responsible decision-making, relationship skills, and social awareness [3]. Educators have recognized arts practices, including visual arts, theater, and dance, as in-school activities that can especially foster these skills [2,4,5]. Children may gain exposure to these activities outside of the academic year through summer programs. 

Understanding the psychological protective factors of SED is important in addressing the issue of declining youth mental health [1]. SED is thought to decrease the rate of mental illnesses such as anxiety and depression through the ability to regulate one's emotions [1]. This emotional regulation also leads to better adaptation in social relationships, demonstrated by increased prosocial behaviors and decreased aggression and related behavioral problems [1]. Additionally, inadequate social support due to a lack of strong social relationships contributes to the development of psychological problems in pediatric patients [6,7]. In addition to benefiting mental health, SED is also associated with increased rates of academic success [1]. Hence, providing children and adolescents with more opportunities for SEL can have long-lasting benefits on their well-being, including their success in school and mental wellness.

More research needs to be done on the relationship between SED and participation in a summer camp program. The learning of social-emotional skills in the summer camp setting, specifically in an arts summer camp, has yet to be explored by current research. The objective of this study is to determine the potential for SED in a community-based art day camp from the summers of 2021-2023 by comparing a qualitative thematic analysis of the art camp's curriculum to the current research regarding the opportunities for SEL in arts education and the opportunities for SEL in summer camps.

This article was previously presented as a poster at the 2024 Florida Atlantic University (FAU) Charles E. Schmidt College of Medicine Medical Student Research and Scholarship Day on February 23, 2024.

## Materials and methods

Camp setting 

The camp took place at a community art center surrounded by a 30.11-acre park with nature trails, play equipment, and open fields. The program ran seven weeks over the summer months. Camp sessions ran four days a week for six hours per day. Camp participants were divided into groups of no more than 10 children, and children stayed with the same group throughout the summer. Camp participants were ages 6-12 years old, counselors ranged from 16 to 21 years old, and counselors-in-training were ages 13-15 years old. Camp staff were hired based on their background experience or personal interest in the arts. Staff underwent a week-long training program, including a first aid course. As camp directors, we oversaw the staff, curriculum, and schedule.

Our camp program was dedicated to the arts and included instruction in various art mediums, including ceramics, collage, painting, drawing, fiber arts, watercolor, oil pastel, origami, poetry, papier-mâché, and chalk drawing. Each day, there were one to three assigned projects, challenging campers to navigate new techniques and materials with their peers. Camp participants engaged in performing arts, including dance, theater, singing, and improv. Camp participants also played outdoor games, went on hikes, visited the local beach, and went on various field trips, including a trip to a professional theater production.

Qualitative thematic analysis

To analyze the curriculum, the Braun and Clarke methodology was used to perform a step-by-step qualitative thematic analysis [8]. An inductive analysis approach was taken to ensure data coding was reflective of the raw data without predetermined classification. The methodology included the following phases: (1) familiarization with the data; (2) generating initial codes; (3) searching for themes; (4) reviewing themes; (5) defining and naming themes; and (6) producing the report [8]. 

Phase 1 consisted of researchers KL and CF repeatedly rereading the summer camp curriculum. The curriculum detailed timestamped activities from each of the 26 days from the seven weeks of the program. The curriculum included the daily plans for the camp activities and projects, including the assigned location and staff members. In phase 2, authors sorted systematically through the data set and identified repetitive patterns to create an initial set of codes grouped by their similarities. In phase 3, the coded curriculum data was organized into candidate themes or subtheme groups by combining data points with common features. For example, activities that included a component of dance were grouped into a single subtheme. Phase 4 involved a review of candidate themes. Themes were adjusted, combined, and divided as necessary. Researchers KL and CF with different backgrounds (MS3 and MAT, respectively) completed the analysis independently to promote different perspectives from curriculum experts. Phase 5 involved a review of the initial coded data to ensure theme categories were labeled to accurately represent the curriculum content. In phase 6, the results were recorded with detailed descriptions of the thematic analysis. This thematic analysis provided a way to determine the themes of the camp curriculum and quantify the days spent in the various activities at camp. Identifying these themes enabled the recognition of opportunities for SED.

Literature search

The Arksey and O'Malley methodology was used for a literature search [9]. This methodology consisted of five steps: (1) identify a research question; (2) search for relevant studies; (3) select studies relevant to the research question; (4) chart the data; and (5) collate, summarize, and report the results. The research question identified for this study was as follows: How does school-aged children's participation in a summer art camp lead to SED? Search terms included social-emotional learning, social-emotional development, art, and camp. The electronic database used was PubMed, and screening of the articles was carried out by the first author. The articles included peer-reviewed studies addressing SED or SEL and art or camp. Studies were excluded that discussed interventions targeted at a pre-existing disease state, focused on older adult populations, or did not focus on SED, SEL, art, or camp. Relevant studies were extracted and analyzed.

Analysis using the CASEL framework 

Finally, the CASEL framework was used to determine the opportunities for SEL in the selected research studies [3]. The results of the qualitative thematic analysis were compared to the interventions carried out in the selected studies to identify the presence of components of the CASEL framework within the camp curriculum.

## Results

Qualitative thematic analysis

The qualitative thematic analysis resulted in the identification of three major themes: art projects, performance, and outdoor activities. Within these major themes, there were clear subthemes. The number of days subtheme codes appeared in the curriculum was counted and the percent of camp days these activities took place was calculated as shown in Table 1.

**Table 1:** Qualitative thematic analysis of camp curriculum

Themes	Subthemes	n (days)	%
Performance	Dance	19	73
	Theater	12	46
	Singing	23	88
Outdoor activities	Unstructured play	26	100
	Hikes	8	31
	Beach trips	10	38
	Group games	24	92
Art projects	Collaborative art projects	5	19
	Individual art projects	22	85
	Presentation of artwork	7	27

The theme of art projects includes the subthemes of collaborative art projects, individual art projects, and presentation of artwork. The theme of art projects encompasses any kind of art project campers created in camp, including all mediums listed in methods. Campers participated in some collaborative art projects (n=5, 19%), in which they worked with other campers to create one final piece of artwork. Many other projects were individual art projects (n=22, 85%), which they completed by themselves. The final subtheme, presentation of artwork (n=7, 27%), refers to campers discussing their art with others or presenting it to a group. There were multiple instances, including the end-of-summer showcase, where campers shared their art in a group setting. 

The theme of performance includes the subthemes of dance, theater, and singing. Performance at camp meant any kind of performance art activity. The subtheme of dance (n=19, 73%) indicates dance rehearsals, classes, improvisation, and choreography. Campers worked toward an end-of-summer theater production in which each child had a speaking role. The theater subtheme (n=12, 46%) included rehearsals for this production, as well as opportunities to learn and refine theater performance skills. The subtheme of singing (n=23, 88%) represented group singing of various camp songs.

The theme of outdoor activities includes the subthemes of unstructured play, hikes, beach trips, and group games. The subtheme of unstructured play (n=26, 100%) accounts for the time that campers played outdoors with each other without the direct guidance of a counselor. Hikes (n=8, 31%) included nature walks in the nearby woods. The subtheme of beach trips (n=10, 38%) accounts for the biweekly beach trips, during which campers swam and played in the sand. The subtheme of group games (n=24, 92%) encompasses the daily athletic group games, such as tag, played during the camp day.

Analysis using the CASEL framework

**Figure 1:**
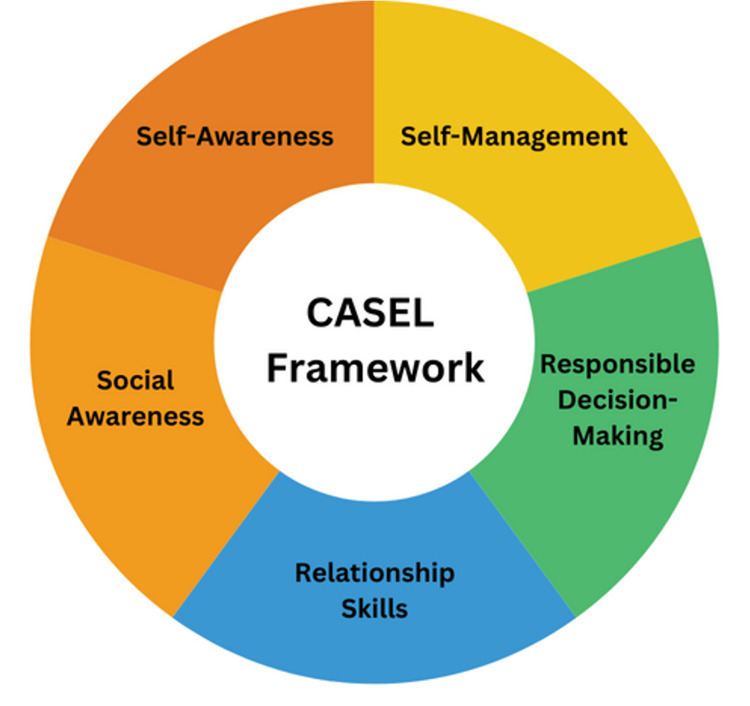
CASEL framework for social-emotional development CASEL: Collaborative for Academic, Social, and Emotional Learning

Considering our own summer art camp in terms of the CASEL framework depicted in Figure 1, we compared the themes resulting from the qualitative thematic analysis with the existing research found during the literature review. The comparison demonstrated that the camp provided ample opportunities for campers to develop social-emotional skills.

Self-Awareness 

There is a relationship between theater practices and the CASEL framework component of self-awareness. The theater served as a major component of our camp, taking place 46% of the days in the camp curriculum. At camp, children had to practice self-awareness by identifying the level of role difficulty they could handle in a theater performance. Per our literature review, a program for previously incarcerated black youth at an alternative school conducted a poetry and theater-based intervention that showed improvement in the self-awareness component of SED [10]. In addition to theater, poetry, creative writing, and self-reflective drawing assignments challenged our campers' self-awareness in a similar way to the intervention practiced in the alternative school [10]. The varied camp activities helped campers develop self-awareness, as they recognized what disciplines they were interested in. 

*Self-Management* 

Summer camp practices have been shown to contribute to the self-management component of SED [11]. More specifically, a two-week summer camp experience was associated with a measured increase in emotional control [11]. In this study, the summer camp included traditional group activities, games, and hikes in nature, all of which were part of the daily curriculum at our summer camp [11]. Group games occurred on 92% of camp days while hikes occurred on 31% of camp days. These camp activities enabled campers to practice self-management while interacting in complex environments and scenarios.

Responsible Decision-Making

The summer camp group dynamic facilitates CASEL's responsible decision-making component. In one study, art sessions followed by a discussion of the art was associated with an increase in responsible decision-making [12]. Our campers discussed art and reflected on their own art in the creation of their final portfolio displayed at the end-of-summer showcase. Twenty-seven percent of camp days had a component of art presentation. Furthermore, biweekly trips to the local beach, occurring on 38% of camp days, allowed campers to move between any of the staffed beach areas but stay with an assigned peer partner. Campers made responsible decisions when they compromised with their partners, maintained awareness, and chose to stay within the appropriate areas. These responsible decisions required emotional self-control, which is shown to be fostered in the collaborative camp environment [11]. 

Relationship Skills

The camp free time yielded benefits in campers' relationship skills. Camp participants had at least one hour of free time per day, during which they engaged in independent play, not facilitated by an adult. Staff were available for conflict resolution and emotional support, but campers were encouraged to navigate peer relationships without staff intervention. One study showed that the combination of caring group leaders and a collaborative peer environment resulted in a significant increase in participant empathy and emotional self-control [11]. Structured camp activities also played a role in the relationship skills component of SED. One paper outlined the benefits of dance, noting the role of interpersonal coordination and neural synchrony in social-emotional skills [13]. Participating in frequent dance classes and performances on 73% of camp days likely contributed to the interpersonal synchrony and non-verbal communication learned through dance.

Social Awareness

As established, painting was a common activity in our camp curriculum. The medium was used frequently in both individual artwork (n=22, 85%) and collaborative artwork (n=5, 19%). Exposure to joint painting in school-aged children led them to make positive conclusions about the coordinated, collaborative art practice [14]. The multiple collaborative art projects provided campers with opportunities to have positive experiences with coordinated art practices. For larger projects taking place on 19% of camp days, such as painting the theater set, campers painted together, thereby practicing joint painting as described in the study [14]. These settings prompted the practice of social awareness skills as campers learned how to work together and respond to their peers' art in a compassionate manner. Finally, being part of a camp environment as a whole is shown to increase empathy [11]. A similar camp environment was built in our camp through the inclusion of free time outdoors (n=26, 100%), group games (n=24, 92%), and hikes (n=8, 31%) all of which fostered a collaborative environment.

## Discussion

As we look for ways to support the mental health of school-aged children, we should not overlook the possibilities for SED that summer camps provide. This study seeks to identify the specific opportunities for SED that our arts summer camp provides to campers, whether through painting, hiking, or singing. Recognizing where the potential opportunities for SED lie in these programs is a valuable step in determining the social and emotional benefits a summer art camp can have for a child. Our qualitative thematic analysis resulted in the identification of three major themes: art projects, performance, and outdoor activities. The literature analyzed supports the SED benefits of the camp activities utilized at our camp. Yet, additional literature presents evidence of the benefits of summer camp for the social-emotional skills of youth. Our summer camp provided a unique environment which combined the facets of an arts education with the experiences of a traditional summer camp.

School-aged children have been shown to benefit in many ways from the summer camp environment. In one study, parents, camp staff, and children reported that camp participants gained confidence, improved self-esteem, social skills, and independence and that these results were maintained six months later [15]. The theater focus which our camp had enabled campers to gain self-awareness and social skills. Performing arts specifically can help children overcome performance anxiety. Because children perform in groups, they also have opportunities to practice compassion and empathy for their peers affected by social anxiety [5]. Performance typically requires portraying a character, which allows children to practice awareness of emotions and recognition of the effect of emotions on behavior [5]. Our campers also gained social awareness when creating individual art projects as well as collaborative art projects. Arts education in school leads to the development of self-management and discipline, interpersonal and relationship skills, and self-expression and identity [5]. One project conducted through Chicago Public Schools supported development in these areas through a variety of artistic pursuits, reporting that the time and practice put into artistic competencies parallel the practice of these developmental skills [5]. Campers developed relationship skills during their unstructured play time, biweekly beach trips, hikes, group games, and collaborative dance practices at camp. One study found that a summer camp program for adolescents increased their ability for perspective-taking and empathic concern [16]. The literature supports the notion that, at summer camps, youth can develop their social and personal skills, such as leadership, emotional regulation, self-esteem, and supportive relationships [17-19]. The thematic analysis demonstrated evidence of opportunities for SED present in our arts summer camp curriculum, emphasizing the many opportunities an arts summer camp can provide for children to promote their SED.

Our findings emphasize that arts summer camps have the potential to benefit the SED of school-aged children and, therefore, should not be overlooked when considering interventions to facilitate SED. Furthermore, our study indicates that more research should be done regarding the opportunities for SED present at summer art camps for school-aged children. With increasing concern around childhood mental health, it is imperative that more research be done into the protective impact of SED and the opportunities for SEL outside of K-12 schooling. 

Limitations

This study is limited by our ability to draw conclusions about the art camp based on the lack of a controlled experimental setting. This study focuses on existing literature compared with the curriculum of a summer art camp, with the participants of this art camp not being studied directly. Another limitation of this study is the difference in demographics between populations in selected research studies. Limited conclusions can be drawn due to the potential differences between our camp population and the varying populations of the cited studies. While all studies focused on youth, variation in age, racial and ethnic groups, and level of art education was present. Lastly, a limitation to the implementation of similar programming on a large scale is that quality summer art camps are not available to everyone, with barriers such as the cost of attendance and the lack of existence in one's community limiting access.

## Conclusions

While it has previously been established that art education in school provides a significant outlet for practicing skills to facilitate SED, more has to be done to establish the relationship between summer art camps and SED. As students in medicine and education, we believe the unique structure of the summer art camp provides the ideal environment for SEL. Art camp as an intervention can benefit children who are struggling in school or have limited access to formal art education, providing the unique opportunity to practice self-expression, friend-making, and self-esteem building. As parents, educators, and healthcare practitioners consider potential interventions to increase children's SED, summer art camps should not be overlooked.
